# Modulation by 17,20S(OH)_2_pD of Fibrosis-Related Mediators in Dermal Fibroblast Lines from Healthy Donors and from Patients with Systemic Sclerosis

**DOI:** 10.3390/ijms23010367

**Published:** 2021-12-29

**Authors:** Monica L. Brown Lobbins, Andrzej T. Slominski, Karen A. Hasty, Sicheng Zhang, Duane D. Miller, Wei Li, Tae-Kang Kim, Zorica Janjetovic, Robert C. Tuckey, Imara-Safi O. Scott, Linda K. Myers, Arnold E. Postlethwaite

**Affiliations:** 1 Department of Pediatrics, University of Tennessee Health Science Center, 50 N. Dunlap, Rm. 461R, Memphis, TN 38103, USA; brownlobbinsrheum20@gmail.com; 2 Department of Medicine, University of Tennessee Health Science Center, 956 Court Avenue, Memphis, TN 38163, USA; imara.hoyte@duke.edu (I.-S.O.S.); acody@uhsc.edu (A.E.P.); 3 Department of Dermatology, University of Alabama at Birmingham, 500 22nd Street South, Birmingham, AL 35294, USA; aslominski@uabmc.edu (A.T.S.); tkkim4567@gmail.com (T.-K.K.); zjanjetovic@uabmc.edu (Z.J.); 4 Comprehensive Cancer Center, University of Alabama at Birmingham, 1824 6th Avenue, Birmingham, AL 35294, USA; 5 Birmingham Veterans Affairs Medical Center, 700 19th Street South, Birmingham, AL 35233, USA; 6 Memphis Veterans Affairs Medical Center, 1030 Jefferson Avenue, Memphis, TN 38104, USA; khasty@uthsc.edu; 7 Department of Orthopaedic Surgery and Biomedical Engineering, University of Tennessee Health Science Center, 1211 Union Avenue, Suite 520, Memphis, TN 38104, USA; 8 Department of Pharmaceutical Sciences, University of Tennessee Health Science Center, 881 Madison Avenue, Memphis, TN 38103, USA; szhang71@uthsc.edu (S.Z.); dmiller@uthsc.edu (D.D.M.); wli@uthsc.edu (W.L.); 9 School of Molecular Sciences, University of Western Australia, 35 Stirling Highway, Perth 6009, Australia; robert.tuckey@uwa.edu.au

**Keywords:** vitamin D, scleroderma, TGF-β1, fibrosis, collagen

## Abstract

We previously demonstrated that the non-calcemic pregnacalciferol (pD) analog 17,20S (OH)_2_pD suppressed TGF-β1-induced type I collagen production in cultured normal human dermal fibroblasts. In the present studies, we examined fibroblasts cultured from the lesional skin of patients with systemic sclerosis (scleroderma (SSc)) and assessed the effects of 17,20S(OH)_2_pD on fibrosis-related mediators. Dermal fibroblast lines were established from skin biopsies from patients with SSc and healthy controls. Fibroblasts were cultured with either 17,20S(OH)_2_pD or 1,25(OH)_2_D_3_ (positive control) with/without TGF-β1 stimulation and extracted for protein and/or mRNA for collagen synthesis and mediators of fibrosis (MMP-1, TIMP-1, PAI-1, BMP-7, PGES, GLI1, and GLI2). 1 7,20S(OH)_2_pD (similar to 1,25(OH)_2_D_3_) significantly suppressed net total collagen production in TGF-β1-stimulated normal donor fibroblast cultures and in cultures of SSc dermal fibroblasts. 17,20S(OH)_2_pD (similar to 1,25(OH)_2_D_3_) also increased MMP-1, BMP-7, and PGES and decreased TIMP-1 and PAI1 expression in SSc fibroblasts. Although 17,20S(OH)_2_pD had no effect on Gli1 or Gli2 in SSc fibroblasts, it increased Gli2 expression when cultured with TGF-β1 in normal fibroblasts. These studies demonstrated that 17,20S(OH)_2_pD modulates mediators of fibrosis to favor the reduction of fibrosis and may offer new noncalcemic secosteroidal therapeutic approaches for treating SSc and fibrosis.

## 1. Introduction

Systemic sclerosis (SSc) (scleroderma) is a chronic disease characterized by extensive fibrosis of the skin and internal organs. The pathogenesis of this disease has been reviewed by Pattanaik, Brown, and Postlethwaite [1] and includes the interplay of autoimmunity, fibroblast activation, increased collagen type I (CI) and III (CIII) deposition, and decreased collagen degradation by metalloproteinases (MMPs). Collagen regulation is tightly controlled by the surrounding extracellular matrix (ECM) [2,3]. TGF-β1 (transforming growth factor beta 1) a key player in fibrogenesis that promotes epithelial to mesenchymal transition (EMT), aids in the transitioning of fibroblast to a myofibroblast phenotype, promotes the conversion of adipocytes to fibroblasts, and promotes fibroblast chemotaxis, collagen synthesis, and plasminogen activator inhibitor (PAI-1) synthesis by fibroblasts [4,5,6,7].

TGF-β1 is effective in inducing fibrogenesis in part because it reduces the expression of matrix metalloproteinase-1 (MMP-1) while increasing the tissue inhibitor of metalloproteinase-1 (TIMP-1) [8,9]. Other factors play a key role in regulating fibrogenesis as well. The bone morphemic protein (BMP-7) is downregulated in fibrosing disorders [10,11], yet it can inhibit CI synthesis and the resulting matrix accumulation [4,12]. PGES (prostaglandin E synthase) can resolve inflammation by activating the release of prostaglandin E2, a direct vasodilator and inhibitor of platelet aggregation and T cell receptor signaling [13,14], although it can be downregulated by TGF-β1 [15,16]. Another contributor to fibrosis, plasminogen activator inhibitor-1 (PAI-1), is induced by TGF-β1, and sustained PAI-1 expression contributes to excessive collagen formation and fibrosis [7,17]. Finally, TGF-β1 induces the expression of glioma-associated oncogene homolog-1 (Gli-1) and -2 (Gli-2) in normal fibroblasts [18,19]. GLI1/GLI2 (the effector molecules of the Hedgehog pathway) promote tissue fibrosis by inducing the fibrotic phenotype of scleroderma.

In recent attempts to find therapeutic interventions for scleroderma, studies have shown that vitamin D (Vit D) deficiency occurs at a higher rate in patients with autoimmune diseases and fibrotic disorders such as SSc [20,21,22]. In SSc, more than 50% of patients have low serum 25-hydroxyvitamin D, and the extent of hypovitaminosis is directly related to the degree of fibrosis, disease severity [23,24], and mortality [25,26]. Dermal thickening, capillary damage, and decreased intestinal absorption contribute to Vit D deficiency [24]. Low serum vit D correlates with increased expression of TGF-β1 [27].

Exposing mesenchymal multipotent cells (MMCs) to 1,25(OH)_2_D_3_ decreases the expression of TGF-β1, CI, and PAI-1 [11]. In localized scleroderma (LS), supplemental treatment with Vit D shows modest positive effects on fibrotic skin lesions [28,29,30]. A randomized control trial (RCT) using paricalcitol, a derivative of Vit D, at 2 µg/d decreases serum levels of TGF-β and IL-6 in kidney transplant recipients [31]. Vit D improved endothelial cell injury and renal fibrosis and the slowed progression of disease in patients with diabetic nephropathy [32]. 

Although these data look promising, current pharmacologic levels are likely not attainable with 1,25 (OH)_2_D_3_ due to its hypercalcemic risk [33,34]. In order to reverse fibrosis, modest doses of Vit D are needed to reach pharmacologic levels and to have an impact on disease morbidity. We have previously published data showing that noncalcemic analogs of Vit D such as 20-hydroxyvitamin D_3_ (20-OHD_3_), a product of CYP11A1 (cytochrome P 450 11A1) action on vitamin D_3_, exhibited potent antifibrogenic activity in vivo and were effective in the inhibition of bleomycin-induced fibrosis [35]. We now study the pregnacalciferol analog 17,20S(OH)_2_pD, which has antifibrotic properties and inhibits TGF-β1-induced net collagen production, without inducing calcemia [36]. The effects are similar to those exerted by the precursors [37]. Functional studies and molecular modeling have demonstrated that it poorly binds to the vitamin D receptor (VDR) [38], indicating an alternative nuclear receptor for this secosteroid with a short sidechain that remains to be identified [37]. Although we previously used normal fibroblasts, we now demonstrate that 17,20S(OH)_2_pD can suppress the total net collagen production and reduces steady-state levels of *Col1A1* mRNA and other mediators of fibrosis in SSc fibroblast cultures. We believe that the results of these studies may help place noncalcemic secosteroidal analogs with a short sidechain as a new class of therapeutic agents for the treatment of SSc.

## 2. Results

### 2.1. 17,20S(OH)_2_pD Suppressed COL1A1 Expression and Total Collagen Production in Healthy Normal and SSc Fibroblasts

**Figure 1:**
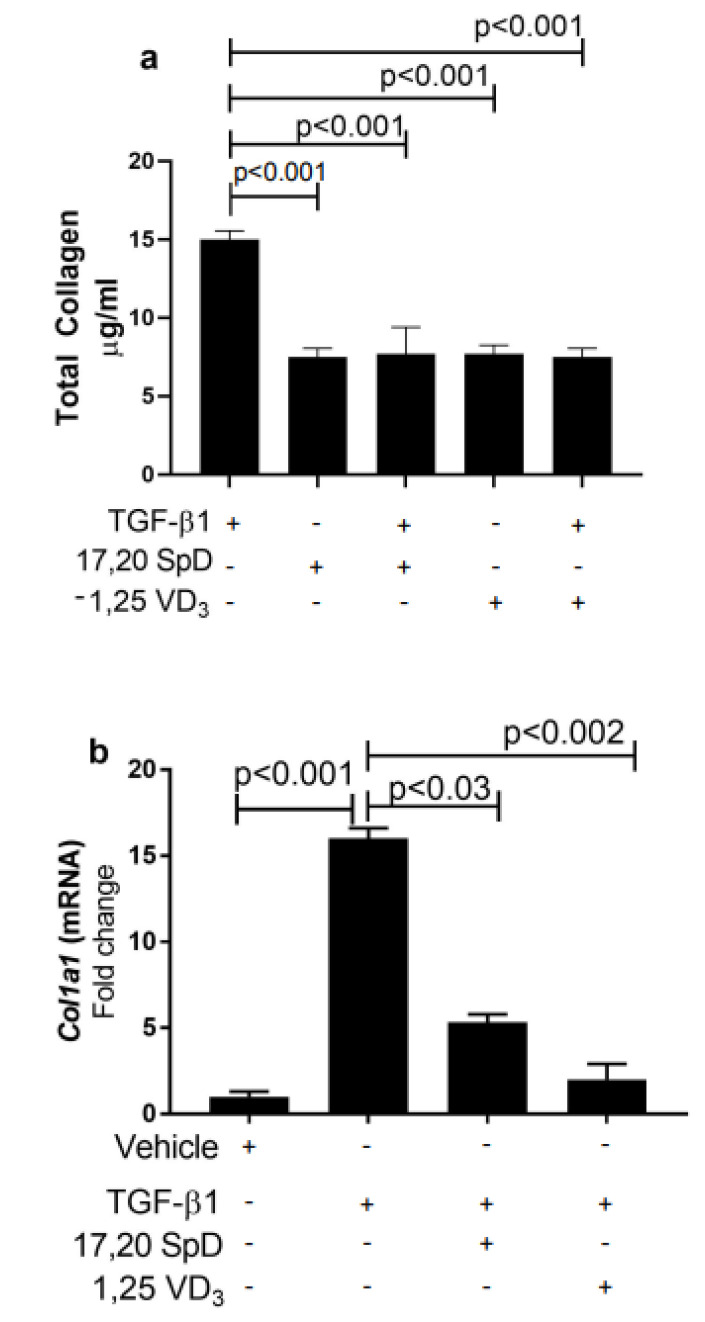
17,20S(OH)_2_pD suppressed total collagen synthesis and COL1A1 in human dermal fibroblasts. SSc human fibroblasts were treated with either 17,20S(OH)_2_pD ^10−8^ M or vehicle (EMEM + 1% stripped FCS + 0.1% BSA with 4 mM HCL (vehicle for TGF-β1) + ETOH (1:10,000 or 1:1 million (vehicle for 17,20S(OH)_2_pD) followed by stimulation with TGF-β1 5 ng/mL for either 24 h for *COL1A1* analysis (**b**) or 48 h for total collagen protein analysis (**a**). mRNA data are expressed as fold change.

Excessive accumulation of collagen has been associated with the thickening and fibrosis of the skin in SSc. We previously showed that 17,20S(OH)_2_pD inhibited TGF-β1-driven collagen synthesis and both type I collagen protein and *Col1A1* mRNA in murine cells and normal human fibroblasts [36]. In this report, we examined total collagen protein synthesis in SSc dermal fibroblasts and found that baseline total collagen production was increased in SSc fibroblasts. On the other hand, culture with 17,20S(OH)_2_pD suppressed the total collagen formation (Figure 1a).

When we examined *COL1a1* expression in SSc dermal fibroblasts, 17,20S(OH)_2_pD decreased *COL1a1* expression when compared to TGF-β1-treated fibroblasts (Figure 1b). These results suggested that 17,20S(OH)_2_pD could suppress TGF-β1-mediated collagen synthesis at both the protein and mRNA level in SSc dermal fibroblasts. 

### 2.2. 17,20S(OH)_2_pD Increases MMP-1 Expression in SSc Fibroblasts

**Figure 2:**
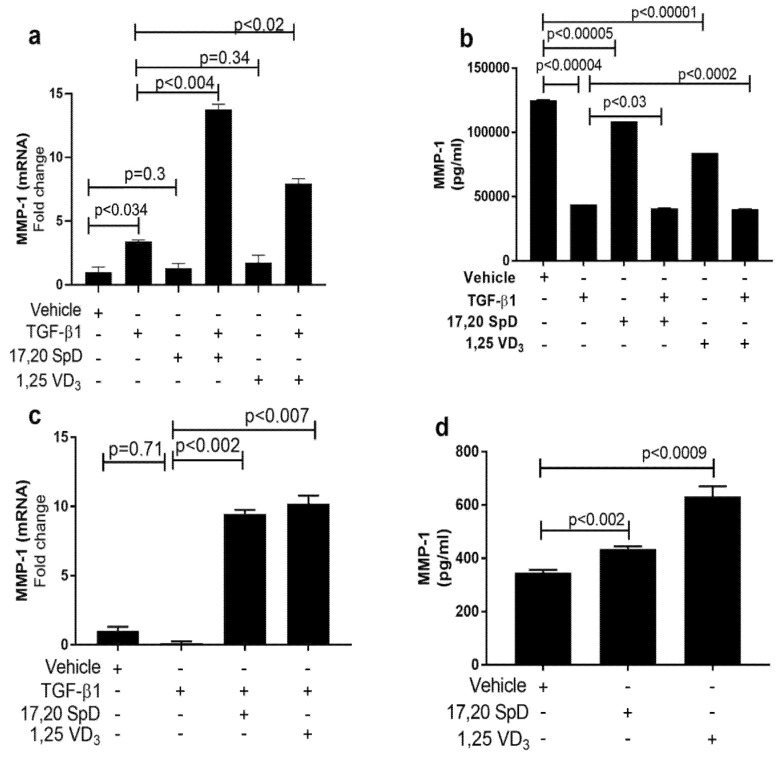
17,20S(OH)_2_pD increased MMP-1 in human dermal fibroblasts. Normal (Panels (**a**,**b**)) and SSc (Panels (**c**,**d)**) dermal fibroblast were treated with either 17,20S(OH)_2_pD ^10−8^ M, 1,25(OH)_2_D_3_ ^10−8^ M, or vehicle (EMEM + 1% stripped FCS + 0.1% BSA with 4 mM HCL (vehicle for TGF-β1) + ETOH (1:10,000 or 1:1 million (vehicle for 17,20S(OH)_2_pD and 1,25(OH)_2_D_3_)), followed by stimulation with TGF-β1 5ng/mL for either 24 h or 48 h and then analyzed for MMP-1 mRNA (fold change) or ELISA (pg/mL).

Excess tissue matrix accumulation in systemic sclerosis (SSc) contributes to fibrosis. It has been suggested that decreased serum levels of matrix metalloproteinases (MMPs) may aggravate this matrix overload. Normal and SSc fibroblasts were cultured with 17,20S(OH)_2_pD to determine the effect on MMP-1. While TGF-β1 slightly increased MMP-1 mRNA and protein expression in normal fibroblasts, the addition of 17,20S(OH)_2_pD to TGF-β increased MMP-1 RNA expression even more (Figure 2a). Normal fibroblasts were not affected by either 17,20S(OH)_2_pD or 1,25(OH)_2_D_3_ (Figure 2b). When we examined SSc fibroblasts, we found that dermal fibroblasts had active TGF-β signaling and spontaneously produced more collagen than cultured fibroblasts from healthy donors [39,40,41]. In SSc fibroblasts, the addition of 17,20S(OH)_2_pD or 1,25(OH)_2_D_3_ increased gene expression and protein synthesis of MMP-1 (Figure 2c,d). These data suggest that both 17,20S(OH)_2_pD and 1,25(OH)_2_D_3_ potentially can alleviate fibrosis by increasing MMP-1. 

### 2.3. 17,20S(OH)_2_pD Decreases TIMP-1 Expression in SSc Fibroblasts

**Figure 3:**
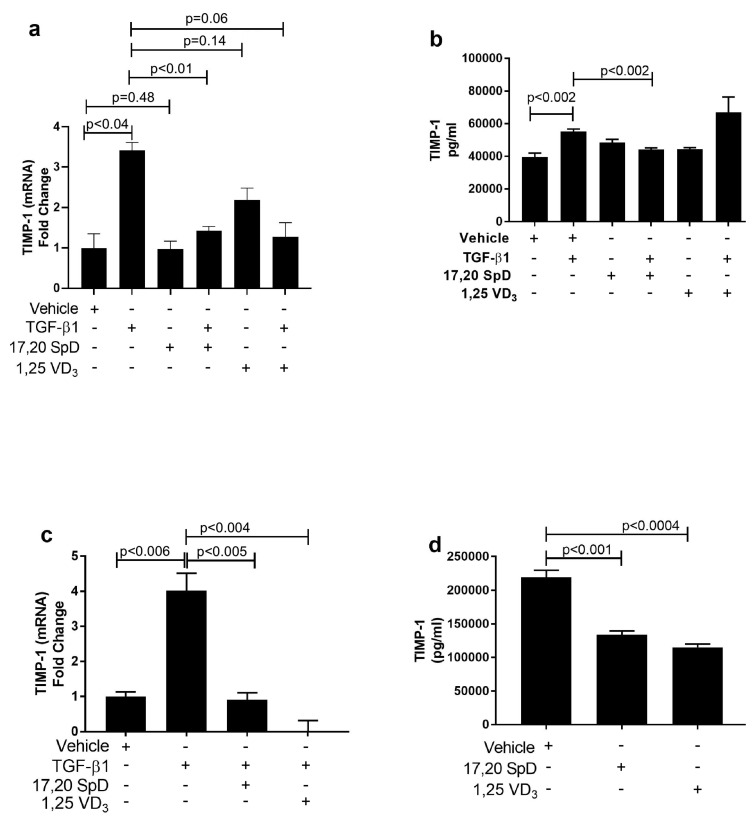
17,20S(OH)_2_pD increased MMP-1 in human dermal fibroblasts. 17,20S(OH)_2_pD decreased TIMP-1 protein synthesis in normal and SSc dermal fibroblasts. Normal (Panels (**a**,**b**)) and SSc (Panels (**c**,**d**)) dermal fibroblast were treated with either 17,20S(OH)_2_pD ^10−8^ M, 1,25(OH)_2_D_3_ ^10−8^ M, or vehicle (EMEM + 1% stripped FCS + 0.1% BSA with 4 mM HCL (vehicle for TGF-β1) + ETOH (1:10,000 or 1:1 million (vehicle for 17,20S(OH)_2_pD and 1,25(OH)_2_D_3_)), followed by stimulation with TGF-β1 5ng/mL for either 24 h or 48 h and then analyzed for TIMP-1 mRNA (fold change) or ELISA (pg/mL). *p* < 0.05 is significant.

Elevated levels of tissue inhibitors of matrix metalloproteinases (TIMPs) may contribute to matrix accumulation in systemic sclerosis (SSc). In normal fibroblasts, TGF-β1 increased the expression of TIMP-1 mRNA and protein. 1,25(OH)_2_D_3_ in the presence of TGF-β1 suppressed TIMP-1 mRNA expression and 17,20S(OH)_2_pD and in the presence of TGF-β1 inhibited protein synthesis of TIMP-1 in normal fibroblasts (Figure 3a,b). In SSc fibroblasts, although basal TIMP-1 mRNA and protein levels were increased, 17,20S(OH)_2_pD and 1,25(OH)_2_D_3_ significantly decreased TIMP-1 protein and mRNA expression in SSc fibroblast lines (Figure 3c,d). These results suggested that 17,20S(OH)_2_pD has anti-fibrogenic properties including the inhibition of collagen synthesis by stimulation of MMP-1 mRNA and protein production and suppression of TIMP-1 protein production in both normal and SSc fibroblasts.

### 2.4. 17,20S(OH)_2_pD Modulates Other Mediators of Fibrosis In Vitro

**Figure 4:**
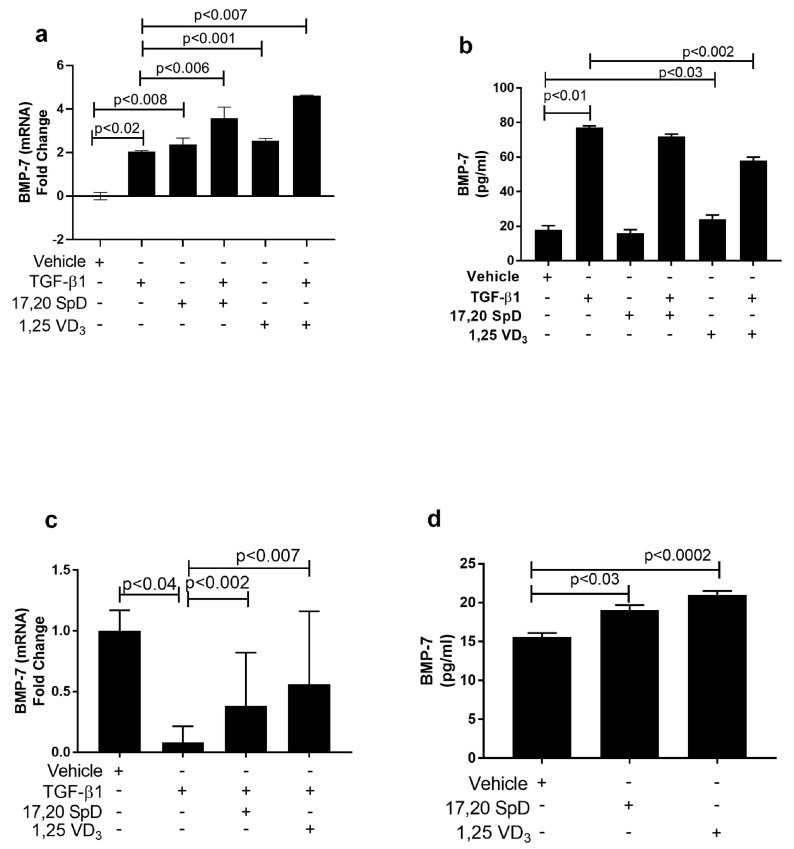
17,20S(OH)_2_pD stimulated BMP-7 synthesis in SSc dermal fibroblasts. Normal (Panels (**a**,**b**)) and SSc (Panels (**c**,**d**)) dermal fibroblast were treated with either 17,20S(OH)_2_pD ^10−8^ M, 1,25(OH)_2_D_3_ ^10−8^ M, or vehicle (EMEM + 1% stripped FCS + 0.1% BSA with 4 mM HCL (vehicle for TGF-β1) + ETOH (1:10,000 or 1:1 million (vehicle for 17,20S(OH)_2_pD and 1,25(OH)_2_D_3_)), followed by stimulation with TGF-β1 5ng/mL for either 24 h or 48 h and then analyzed for BMP-7 by either mRNA (fold change) or ELISA (pg/mL). *p* < 0.05 is significant.

TGFβ-1 is a potent stimulator of collagen synthesis by fibroblasts. On the other hand, even in the presence of TGF-β1, bone morphemic protein (BMP-7) can inhibit CI synthesis and the resulting matrix accumulation. Normal and SSc dermal fibroblasts were cultured with 17,20S(OH)_2_pD to examine its effect on BMP-7, PGES, and PAI-1 expression. Although BMP-7 mRNA expression was slightly increased in TGF-β1-stimulated normal fibroblasts, 17,20S(OH)_2_pD either alone or co-cultured with TGF-β1 further increased BMP-7 mRNA expression. BMP-7 expression was also increased with 1,25(OH)_2_D_3_ alone and in the presence of TGF-β1. Protein levels were not affected by 17,20S(OH)_2_pD in normal fibroblasts (Figure 4a,b). On the other hand, SSc fibroblasts had decreased basal expression of BMP-7, so that the addition of 17,20S(OH)_2_pD significantly increased the expression of BMP-7. A similar increase was seen when fibroblasts were cultured with 1,25(OH)_2_D_3_ (Figure 4c,d). These data suggest that in both normal and SSc fibroblasts, 17,20S(OH)_2_pD either alone or in the presence of TGF-β1 can increase BMP-7 expression, helping alleviate excessive fibrosis.

**Figure 5:**
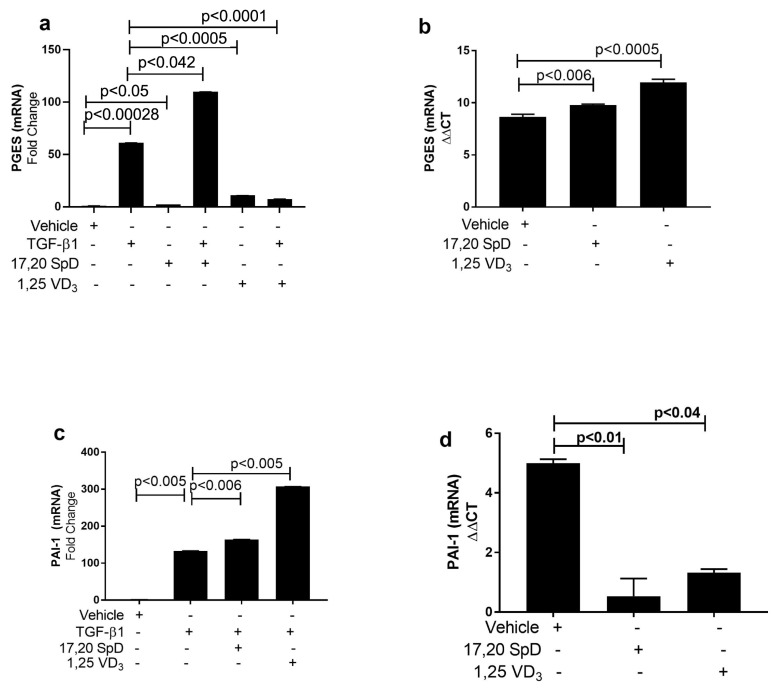
17,20S(OH)_2_pD increases PGES and inhibits PAI-1 mRNA expression in normal and SSc dermal fibroblasts. Normal (Panels (**a**,**c**)) and SSc (Panels (**b**,**d**)) dermal fibroblast were treated with either 17,20S(OH)_2_pD ^10−8^ M, 1,25(OH)_2_D_3_ ^10−8^ M, or vehicle (EMEM + 1% stripped FCS + 0.1% BSA with 4 mM HCL (vehicle for TGF-β1) + ETOH (1:10,000 or 1:1 million (vehicle for 17,20S(OH)_2_pD and 1,25(OH)_2_D_3_)), followed by stimulation with TGF-β1 5 ng/mL for 24 h and then analyzed for PGES or PAI-1 mRNA expression (fold change). *p* < 0.05 is significant.

TGF-β1 ordinarily decreases prostaglandin E2 synthetase (PGES) production. PGES can resolve inflammation because it activates the release of prostaglandin E2, which acts as a direct vasodilator and has antifibrotic effects such as inhibition of fibroblast chemotaxis, proliferation, and collagen formation. In normal fibroblasts, 17,20S(OH)_2_pD together with TGF-β1 increased PGES expression, although there was no significant change in PGES when fibroblasts were co-cultured with 17,20S(OH)_2_pD alone or 1,25(OH)_2_D_3_ alone (Figure 5a). In scleroderma fibroblasts, PGES was induced when cultured with 17,20S(OH)_2_pD if compared to vehicle-treated fibroblasts. PGES expression was also increased in SSc fibroblasts when cultured with 1,25(OH)_2_D_3_ (Figure 5b). These data suggest that in both normal and scleroderma fibroblasts, 17,20S(OH)_2_pD increased PGES synthesis, suggesting that 17,20S(OH)_2_pD may have anti-inflammatory properties.

In inflammatory conditions in which fibrin is deposited in tissues, PAI-1 appears to play a significant role in the progression to fibrosis. Presumably, lower PAI levels would lead to less suppression of fibrinolysis and conversely a more rapid degradation of the fibrin. Sustained PAI-1 expression contributes to excessive collagen formation and fibrosis. We examined the effect of 17,20S(OH)_2_pD on PAI-1 mRNA expression in both normal and SSc fibroblasts. In normal fibroblasts, TGF-β1 increased PAI-1 expression as previously described [7] (Figure 5c). Normal fibroblasts cultured with 17,20S(OH)_2_pD or 1,25(OH)_2_D_3_ had no effect on PAI-1 when compared to vehicle-cultured fibroblasts. In the presence of TGF-β1, both 17,20S(OH)_2_pD and 1,25(OH)_2_D_3_ increased the expression of PAI-1 (Figure 5c). In SSc fibroblasts, PAI-1 expression was increased at baseline. Culturing SSc fibroblasts with 17,20S(OH)_2_pD significantly decreased PAI-1 expression. This same suppressive effect was seen in fibroblasts cultured with 1,25(OH)_2_D_3_ (Figure 5d). These results suggest that 17,20S(OH)_2_pD in the presence of TGF-β1 can effectively inhibit PAI-1 mRNA expression in SSc fibroblasts, leading to less fibrosis.

**Figure 6:**
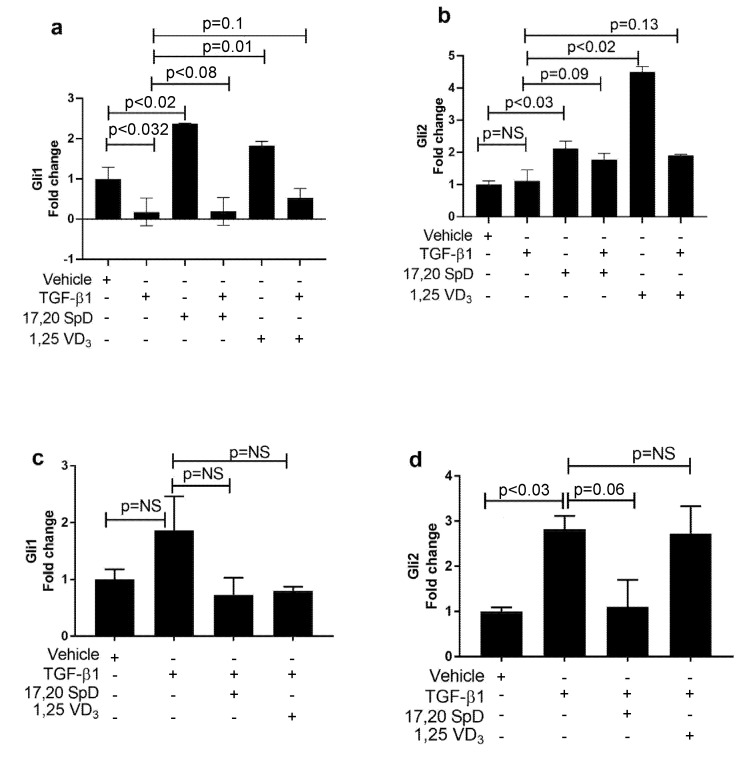
17,20S(OH)_2_pD had no effect on GLi mRNA expression in SSc dermal fibroblasts, but in the presence of TGF-β1 increases Gli2 expression in normal fibroblast. Normal (Panels (**a**,**b**)) and SSc (Panels (**c**,**d**)) dermal fibroblast were treated with either 17,20S(OH)_2_pD ^10−8^ M, 1,25(OH)_2_D_3_ ^10−8^ M, or vehicle (EMEM + 1% stripped FCS + 0.1% BSA with 4 mM HCL (vehicle for TGF-β1) + ETOH (1:10,000 or 1:1 million (vehicle for 17,20S(OH)_2_pD and 1,25(OH)_2_D_3_)), followed by stimulation with TGF-β1 5 ng/mL for 24 h and then analyzed for Gli1 or Gli2 mRNA expression (fold change). NS is not significant. *p* < 0.05 is significant.

The induction of the fibrotic phenotype of scleroderma is mediated through GLI1/GLI2 (the effector molecules of the Hedgehog pathway) to promote tissue fibrosis. We analyzed the effect of 17,20S(OH)_2_pD on GLI1 and GLI2 mRNA expression in both normal and SSc fibroblasts. In normal fibroblasts, the addition of 17,20S(OH)_2_pD increased GLI1 mRNA expression, while the addition of TGF-β1 together with 17,20S(OH)_2_pD abrogated the stimulatory effect of the latter on GLI1 expression. The stimulation of GLI1 expression by 1,25(OH)_2_D_3_ alone did not reach the statistical significance, and concomitant addition of TGF-β1 significantly decreased this expression (Figure 6a). In the presence of TGF-β1, both 17,20S(OH)_2_pD and 1,25(OH)_2_D_3_ increased GLI2 mRNA expression (Figure 6b). When SSc fibroblasts were examined, neither 17,20S(OH)_2_pD, nor 1,25(OH)_2_D_3_ had a significant effect on either GLI1 or Gli2 mRNA expression (Figure 6c,d). Taken together, these data suggest that 17,20S(OH)_2_pD may partially inhibit GLI1 in normal fibroblasts, but is not effective in suppressing this pathway in SSc fibroblasts.

## 3. Discussion

Our results showed that 17,20S(OH)_2_pD suppressed TGF-β1-induced collagen protein production in cultured dermal normal and SSc fibroblasts. Moreover, 17,20S(OH)_2_pD effectively increased MMP-1 synthesis and suppressed TIMP-1 production, both factors allowing 17,20S(OH)_2_pD to enhance collagen degradation and decrease fibrosis. Since PGES and BMP-7 can help alleviate fibrosis, it is significant that in both normal and SSc fibroblasts, 17,20S(OH)_2_pD increased PGES and BMP-7. On the other hand, sustained PAI-1 aggravates fibrosis. 17,20S(OH)_2_pD was able to suppress PAI-1 mRNA expression in both normal and SSc fibroblasts, while it had no effect on Gli1 and Gli2 expression in SSc fibroblasts. Taken together, these results indicate that 17,20S(OH)_2_pD can modulate mediators of the TGF-β1 pathway in vitro, suggesting its potential to have anti-inflammatory properties in vivo and, by implication, anti-sclerotic effects. 

In patients with SSc, dermal thickening, capillary damage, and decreased intestinal absorption contribute to vitamin D deficiency [24]. Vitamin D deficiency correlates with increased expression of TGF-β1 [27]. In vitro studies have shown that 1,25(OH)_2_D_3_ and analogs of vitamin D inhibited the TGF-β effect on collagen synthesis and topical treatment with a vitamin D analogue reduced skin fibrosis [42]. 1,25(OH)_2_D_3_ suppressed TGF-β1-induced collagen synthesis in lung fibroblasts, stellate cells from the liver, and renal cells [43,44]. Exposing mesenchymal multipotent cells (MMCs) to 1,25(OH)_2_D_3_ decreased the expression of TGF-β1, CI, CIII, and PAI-1 and increased BMP-7 expression [11]. In our study, we demonstrated for the first time that the secosteroidal analog with a short side chain, 17,20S(OH)_2_pD, inhibited collagen synthesis, TIMP-1, and PAI-1, while it increased MMP-1, PGES, and BMP-7 expression. In SSc fibroblast, 17,20S(OH)_2_pD suppressed collagen synthesis, TIMP-1, and PAI-1, while it increased the expression of MMP-1, BMP-7, and PGES and had no effect on Gli1 or Gli2 expression. 

TGF-β1 is a major cytokine involved in fibrosing disorders. TGF-β1 increases collagen synthesis in dermal fibroblasts and contributes to the production of extracellular matrix proteins that favor fibrosis [5,45]. SSc fibroblasts express endogenous TGF-β1 [46]. Nephrogenic systemic fibrosis skin biopsies display increased expression of TIMP-1, but little to no MMP-1 [47]. The lack of metalloproteinases contributes to the collagen accumulation that is characteristic of fibrotic disorders [48]. Serum levels of MMP-1 have been shown to negatively correlate with the degree of fibrosis [49,50]. 

TGF-β also modulates the expression of BMP-7, PGES, PAI-1, GLI1, and GLI2, which also contribute to fibrosis [10,11,16,17,18]. BMP-7, a member of the TGF-β superfamily, is an antagonist of TGF-β1 and is downregulated in disorders of fibrosis in the presence of TGF-β1 upregulation [10,11]. BMP-7 has a role in preventing the TGF-induced EMT by decreasing CI synthesis [4,12]. Treatment with BMP-7 has been shown to inhibit renal, liver, and cardiac fibrosis [51,52]. In our study, BMP-7 synthesis was decreased in SSc fibroblasts and in normal fibroblasts cultured with TGF-β1.

Prostaglandin (PG) E_2_ has antiproliferative and antifibrotic properties. Its antifibrotic effects are manifested by the inhibition of fibroblasts’ chemotaxis, proliferation, and collagen formation [53,54,55]. TGF-β1 decreases PGE production [15]. In vitro studies using mesangial cells showed that TGF-β1 increased the proliferation of these mesangial cells in addition to decreasing PGE production. Li and coworkers found that following EMT, PGE_2_ inhibits chemotaxis by increasing the expression of E-prostanoid receptors 2 (EP2) and 4 (EP4) [16]. Prostaglandin E2 synthase (PGES) catalyzes the conversion of prostaglandin to PGE2. Although in SSc fibroblasts, the expression of PGES is mildly upregulated when compared to normal fibroblasts [13,14], we showed that treatment with 17,20S(OH)_2_pD further increased PGES expression in both normal and scleroderma fibroblasts. These data suggested that 17,20S(OH)_2_pD might have anti-inflammatory properties.

Urokinase/tissue-type plasminogen activator (uPA/tPA) and plasmin-dependent MMPs function in maintaining tissue homeostasis by degrading proteins of the ECM. uPA/tPA converts latent TGF-β and pro-MMP to their active forms [17]. PAI-1, a serine protease inhibitor, is a potent inhibitor of uPA/tPA by blocking uPA/tPA activation and plasmin-dependent MMP activation [17]. Although PAI-1 is a major contributor to efficient wound healing due to its ability to protect ECM proteins from proteolytic degradation, under pathologic conditions such as SSc, increased levels of PAI-1 contribute to collagen accumulation and increased expression of ECM proteins. TGF-β1 is a known inducer of PAI-1 [7]. Increased PAI-1 levels in SSc fibroblasts are due to increased TGF-β1 expression, SMAD2/3 activation, and decreased expression of inhibitory SMAD7. Sustained PAI-1 expression contributes to excessive collagen accumulation, leading to fibrosis [17]. Our results showed that in normal fibroblasts, TGF-β1 increased PAI-1 expression, while the addition of 17,20S(OH)_2_pD decreased the expression of PAI-1. In SSc fibroblasts, baseline expression of PAI-1 was increased, and 17,20S(OH)_2_pD decreased PAI-1 expression. Our results suggested that 17,20S(OH)_2_pD in the presence of TGF-β1 could effectively inhibit PAI-1 mRNA expression in both normal and SSc fibroblasts. 

GLI1 and GLI2 are transcription factors in the Sonic Hedgehog (SHH) pathway. GLI1 and GLI2 have important roles in cell development and differentiation and are involved in myofibroblast formation. Lung tissues from patients with idiopathic pulmonary fibrosis have increased expression of GLI1 and GLI2 when compared to normal lung tissues. Lung epithelium and fibroblasts from these patients expressed increased GLI1 levels, while alveolar cells expressed increased GLI2 in a nuclear distribution. Fibroblasts that were treated with SHH had increased collagen synthesis and gene expression [56]. TGF-β1 induces the expression of GLI1 and GLI2 in normal fibroblasts and keratinocytes [18,19] and induces the expression of SHH and GLI1 in lung epithelial cells [18]. In our study, normal fibroblasts behaved differently from SSc fibroblasts. GLI1 mRNA expression decreased with TGF-β1 in normal fibroblasts, and culture with 17,20S(OH)_2_pD increased Gli1 mRNA expression. The addition of TGF-β1 with 17,20S(OH)_2_pD increased GLi2 expression, but not GL1 in normal fibroblasts. On the other hand, 17,20S(OH)_2_pD had no significant effect on either GLI1 or Gli2 mRNA expression in SSc fibroblasts. Taken together, these data demonstrate that 17,20S(OH)_2_pD affects normal and SSc fibroblasts differently in regard to its effect on Gli1 and Gli2. However, a precise mechanism of action remains unclear, since the corresponding nuclear receptor for 17,20S(OH)_2_pD remains to be identified. It must be noted that CYP11A1 hydroxyderivatives can act on alternative nuclear receptors including retinoic acid orphan receptors (RORs) [57], the aryl hydrocarbon receptor (AhR) [58], and liver X receptors (LXRs) [59], as well as their increased selectivity on the VDR requires hydroxylation on C1α [60]. Although 17,20S(OH)_2_pD can act as a weak agonist on LXRα and LXRβ [59], the high affinity receptor for hydroxysecosteroids with a short side chain remains to be defined.

Limitations of this study: Our study highlights a potential role for 17,20S(OH)_2_pD in the treatment of fibrosis. Our data are limited by the number of fibroblasts analyzed and treated with 17,20S(OH)_2_pD. We understand that differences in patient populations could alter the response of 17,20S(OH)_2_pD on collagen, TGF-β1, and other mediators of fibrosis. We understand that other pathways not related to TGF-β1 may be involved in 17,20S(OH)_2_pD suppression of collagen levels. Importantly, the nuclear receptor through which 17,20S(OH)_2_pD mediates its phenotypic effects remains to be defined, a future challenge to our laboratories. This report has discrepancies between mRNA levels and protein levels of MMP-1 and BMP7. Unfortunately, in some instances, the mRNA expression differs from protein expression levels due to: (a) complicated post-transcriptional mechanisms; (b) differences in the in vivo half-lives of proteins; (c) error and noise in both protein and mRNA experiments. Moreover, short-chain analogs of vitamin D, such as 17,20R(OH)_2_pD and 17,20S(OH)_2_pD, differ structurally from 1,25(OH)_2_D3. The biochemical differences in the side chains may explain their differing effects on the induction of calcemia and varying levels of PGES. 

## 4. Materials and Methods

### 4.1. Secosteroids

1,25(OH)_2_D_3_ was obtained from Sigma-Aldrich, Co. LLC (St. Louis, MO, USA). Secosteroid: 17,20S(OH)_2_pD was synthesized as previously described [61]. For the tissue culture experiments, 1,25(OH)_2_D_3_ and 17,20S(OH)_2_pD were dissolved in absolute ethanol and diluted with Eagle’s minimal essential medium (EMEM) containing 1% charcoal-stripped fetal calf serum (FCS) at a stock concentration of 10^−5^ M for each. 

### 4.2. Cell Lines and Culture

This study was conducted and approved by the University of Tennessee Health Science Center (UTHSC) Memphis Institutional Review Board (#07-08719). The Declaration of Helsinki protocols were followed, and normal healthy donors and SSc patients gave their written informed consent. Adult patients (ages 18–70 y) who met the American College of Rheumatology criteria for the diagnosis of SSc were used in this study. Punch biopsies from the lesional skin of patients with diffuse cutaneous systemic sclerosis (dcSSc) were taken from the volar forearm surface. Punch biopsies from the volar forearm surface were taken from normal healthy donors. Explanted biopsy specimens were allowed to grow into fibroblasts for 2–3 wk in Eagle’s minimal essential medium (EMEM) (Corning Cellgro, Manassas, VA, USA) with 9% fetal calf serum (FCS), 100 µL/mL penicillin, 100 µg/mL streptomycin, and amphotericin B 1 µg/mL and ascorbic acid (50 µg/mL) (Thermo Fisher, Waltham, MA, USA) as previously described by Brown Lobbins et al. [36,62]. In total, there were 5 normal fibroblast lines and 5 SSc lines. All were age, gender, and ethnicity matched. Each study used 3 cell lines per group, and passages 3–7 were used.

Fibroblasts were cultured and maintained in EMEM with 10% FCS, penicillin (100 units/mL), streptomycin (100 mg/mL), and 2 mM L-glutamine at 37 °C in a 5% CO_2_ humidified atmosphere. Fibroblasts between 10 and 16 passages were used for the experiments. Prior to the experiments, the fibroblast medium was changed to EMEM supplemented with 10% charcoal (Sigma-Aldrich, St. Louis, MO, USA)-stripped fetal calf serum (Atlanta Biologics, Flowery Branch, GA, USA) for 72 h. After a 72 h incubation period, the medium was changed to 1% charcoal-stripped FCS for 24 h. Following the 24 h incubation period, fibroblasts were incubated with 17,20S(OH)_2_pD or 1,25(OH)_2_D_3_ at either 10^−8^ M or 10^−9^ M or ethanol (ETOH) vehicle at 1:10,000 or 1:1 million dilution to control for final ETOH concentration in the 10^−8^ M and 10^−9^ M 17,20S(OH)_2_pD and 1,25(OH)_2_D_3_ preparations in culture. Fibroblasts were allowed to incubate under these conditions for a 2 h period before stimulation with TGF-β1 (R&D Systems, Minneapolis, Minnesota USA) dissolved in 0.1% bovine serum albumin (BSA) in 4 mM hydrochloric acid (Gibco, Life Technologies, Waltham MO, USA) to a final concentration of 5 ng/mL or BSA/HCL diluted 1:2000 to control for the final BSA-HCL in TGF-β1 concentration. Fibroblasts were cultured under these conditions either for an additional 24 h for total RNA extraction and 48 h for the analysis of total collagen or protein synthesis, respectively. 

### 4.3. Quantitative Real-Time PCR

Total RNA was extracted from fibroblast monolayers using TRIzol reagent (Invitrogen-Thermo Fisher Scientific, Waltham, MA, USA) and reversed transcribed into cDNA using reverse transcriptase (Life Technologies-Applied Biosystems (ABI), Grand Island, NY, USA), for real-time -polymerase chain reaction (RT-PCR). RNAs were quantified using NanoDrop-2000 (Thermos, Franklin, NJ, USA). Fifteen nanograms of total RNA were used for these experiments. The TaqMan gene assay for human COL1A1 (Cat. No. HS01076777), MMP-1 (Cat. No. HS00899658), TIMP-1 (Cat. No. HS00171558), PAI-1 (Cat. No. HS01126606), BMP-7 (Cat. No. HS00233476), PGES (Cat. No. HS00153133), Gli1 (Cat. No. Hs01110766), Gli2 (Cat. No Hs01119974), and beta actin (Cat. No. HS01060665) (Applied Biosystems Life technologies, Grand Island, NY, USA) was used as a housekeeping gene. SDS Version 2.3 software was used to run the PCR, and the analysis was obtained with RQ manager (Life Technologies-ABI). 17,20S(OH)_2_pD and 1,25(OH)_2_D_3_ were normalized to β-actin (Applied Biosystems Life Technologies, Grand Island, NY, USA) mRNA expression levels. Data for 17,20S(OH)_2_pD- and 1,25(OH)_2_D_3_-treated fibroblast cultures are shown as fold change normalized to an endogenous reference (BSA + ETOH) vehicle control.

### 4.4. Protein Analysis

The supernatants from cultured fibroblasts were analyzed for protein concentrations using ELISA for MMP-1 (Cat No. DY901, R&D systems; Minneapolis, MN, USA), TIMP-1 (Cat No. DTM100, R&D systems; Minneapolis, MN, USA), and BMP-7 (Cat No. DBP700, R&D systems; Minneapolis, MN, USA). 

### 4.5. Analysis of Total Collagen

Fibroblasts monolayers were extracted for total collagen levels using the Sircol Collagen Assay (Biocolor Ltd., Carickfergus, Northern Ireland, UK). Before performing these experiments, we compared the data generated by Sircol to other colorimetric methods to measure hydroxyproline and found the results to be comparable.

### 4.6. Statistical Analyses

The calculations to determine the statistical significance of the tests were carried out using the programs SAS and GraphPad Prism 4. Depending on the data, a one-way ANOVA or Student’s *t*-test analysis was performed. The comparison of the mean variable values with a distribution significantly different from normal in two unrelated groups was performed using the Mann–Whitney test, while in more than two unrelated groups, using the Kruskal–Wallis test. *p* ≤ 0.05 was considered statistically significant.

## 5. Conclusions

We showed that 17,20S(OH)_2_pD is able to suppress TGF-β1-induced collagen protein production in cultured dermal normal and SSc fibroblasts. 17,20S(OH)_2_pD inhibits collagen synthesis by effectively modulating mediators of the TGF-β1 pathway. The key players we studied were BMP-7, PAI-1, and PGES. These changes lead to altered metalloproteinase synthesis and the degradation of collagen. Although 17,20S(OH)_2_pD and 1,25(OH)_2_D3 are similar molecules, they do have important critical differences, such as their ability to induce hypercalcemia. The differential effects on TIMP-1 in normal fibroblasts appears to be another difference between the two secosteroids. Further work will be needed to determine why 1,25(OH)_2_D3 has such differing effects on normal and SSc dermal fibroblasts. Possibilities include the fact that 17,20S(OH)_2_pD has a shortened side chain and is lacking a hydroxyl group on C1α. Furthermore, it likely targets a different nuclear receptor than the VDR, which remains to be identified. In summary, our study supports that 17,20S(OH)_2_pD modulates mediators of fibrosis wherein a balance will favor the reduction of fibrosis and may offer a new therapeutic approach for treating SSc and other disorders of fibrosis.

## Data Availability

The datasets used and/or analyzed during the current study are available from the corresponding author upon reasonable request.

## Abbreviations

1:25(OH)_2_D_3_	1:25-dihydroxyvitamin D3
17,20(OH)_2_pD	17,20-dihydroxypregnacalciferol
BMP-7	Bone morphogenic protein 7
Gli1	Glioma-associated oncogene homolog 1
Gli2	Glioma-associated oncogene homolog 2
MMP-1	Metalloproteinase-1
PAI-1	Plasminogen activator inhibitor-1
PGES	Prostaglandin E2 synthetase
TIMP-1	Tissue inhibitor of metalloproteinase-1
TGF-β1	Transforming growth factor-β1
